# Challenge of prostate MRI segmentation on T2-weighted images: inter-observer variability and impact of prostate morphology

**DOI:** 10.1186/s13244-021-01010-9

**Published:** 2021-06-05

**Authors:** Sarah Montagne, Dimitri Hamzaoui, Alexandre Allera, Malek Ezziane, Anna Luzurier, Raphaelle Quint, Mehdi Kalai, Nicholas Ayache, Hervé Delingette, Raphaële Renard-Penna

**Affiliations:** 1 Academic Department of Radiology, Hôpital Pitié-Salpétrière, Assistance Publique des Hôpitaux de Paris, Paris, France; 2 Academic Department of Radiology, Hôpital Tenon, Assistance Publique des Hôpitaux de Paris, Paris, France; 3 Inria, Epione Team, Université Côte D’Azur, Sophia Antipolis, Nice, France; 4 Sorbonne Universités, GRC n° 5, Oncotype-Uro, Paris, France

**Keywords:** Prostate, MRI, Segmentation, Zones, Atlas

## Abstract

**Background:**

Accurate prostate zonal segmentation on magnetic resonance images (MRI) is a critical prerequisite for automated prostate cancer detection. We aimed to assess the variability of manual prostate zonal segmentation by radiologists on T2-weighted (T2W) images, and to study factors that may influence it.

**Methods:**

Seven radiologists of varying levels of experience segmented the whole prostate gland (WG) and the transition zone (TZ) on 40 axial T2W prostate MRI images (3D T2W images for all patients, and both 3D and 2D images for a subgroup of 12 patients). Segmentation variabilities were evaluated based on: anatomical and morphological variation of the prostate (volume, retro-urethral lobe, intensity contrast between zones, presence of a PI-RADS ≥ 3 lesion), variation in image acquisition (3D vs 2D T2W images), and reader’s experience. Several metrics including Dice Score (DSC) and Hausdorff Distance were used to evaluate differences, with both a pairwise and a consensus (STAPLE reference) comparison.

**Results:**

DSC was 0.92 (± 0.02) and 0.94 (± 0.03) for WG, 0.88 (± 0.05) and 0.91 (± 0.05) for TZ respectively with pairwise comparison and consensus reference. Variability was significantly (*p* < 0.05) lower for the mid-gland (DSC 0.95 (± 0.02)), higher for the apex (0.90 (± 0.06)) and the base (0.87 (± 0.06)), and higher for smaller prostates (*p* < 0.001) and when contrast between zones was low (*p* < 0.05). Impact of the other studied factors was non-significant.

**Conclusions:**

Variability is higher in the extreme parts of the gland, is influenced by changes in prostate morphology (volume, zone intensity ratio), and is relatively unaffected by the radiologist’s level of expertise.

**Supplementary Information:**

The online version contains supplementary material available at 10.1186/s13244-021-01010-9.

## Key Points

- Variability of prostate segmentation is higher in the extreme parts of the gland.
- Variability of prostate segmentation increases with prostate volume.
- Variability of zonal prostate segmentation is not substantially affected by the interpreting radiologist’s level of expertise.

## Introduction

Segmentation of prostate MRI plays a crucial role in many existing and developing clinical applications, including prostate cancer staging and treatment planning. Prostate segmentation of the whole gland has to be performed frequently in routine clinical practice mainly for MRI-US fusion biopsy or radiotherapy planning, and can be used to evaluate prostate volume for PSA density calculation. Manual segmentation of the prostate is usually performed on T2-weighted (T2W) images by contouring the prostate in a slice-by-slice manner using either the axial, sagittal, or coronal views, or a combination of different views. It is extremely time-consuming, tedious, and prone to inter and intra-observer variation due to the large variability in prostate anatomy across patients [1], and prostate gland intensity heterogeneity.

There is a real need to develop automatic methods that offer robust and accurate prostate segmentation. The majority of previous works involving manual segmentation was initially focused on whole gland segmentation [2–4] and little attention was paid to segmenting the internal structure of the prostate.

Over the years the most common indication for prostate MRI transitioned from merely staging prostate cancer (PCa) to detecting it [5], and MRI is now recommended in biopsy-naive patients [6, 7]. The PI-RADS scoring system [8, 9], designed to detect PCa, is based on the internal structure of the prostate, divided into four histological zones called the peripheral (PZ), transitional (TZ), central (CZ) zones and the anterior fibromuscular stroma (AFMS) [10].

Thus, the focus for automatic prostate segmentation went from whole gland segmentation to zonal segmentation of the gland [11, 12], which is now necessary for the development of AI algorithms for prostate cancer detection.

The quality of a segmentation is evaluated by comparing it to a reference segmentation, often designated as ground truth. Manual delineation of the prostate gland performed by human experts (radiologists or radiation oncologists) is the main approach to generate ground truth. Several teams [13–15] have trained their models on the prostate MRIs and the relative manual ground truth annotation available from the PROMISE12 challenge [4], based on the final segmentation of a single expert reader [4]. Very few studies have systematically investigated inter-reader variability in zonal segmentation due to reader expertise [16], anatomical or disease-induced variations in the prostate aspect, or technically-induced variability in the image acquisition. There are no current guidelines for prostate zonal segmentation. However, uncertainties in contouring can be an issue when performing targeted biopsies, or for treatment planning, and for the development of automated PCa detection algorithms.

Hence, the purpose of our study was to investigate the inter-reader variability when delineating prostate zonal anatomy, and the impact of reader expertise, variations in prostate anatomy, cancer-induced modifications, and, for a subgroup of patients, technical differences in image acquisition.

## Material and methods

### Dataset

This work was supported by the Health Data Center of the AP-HP (Assistance Publique-Hôpitaux de Paris) and was approved by our joint institutional review boards. Data were extracted from the Clinical Data Warehouse of the Greater Paris University Hospitals. We compiled a cohort of 40 patients from a larger cohort/dataset (in house, *n* = 150) of treatment-naive patients who underwent a prostate MRI before the first round of biopsy for clinical suspicion of PCa between October 2013 and July 2019. This dataset included patients fulfilling the inclusion criterion for clinical indication of prostate MRI for suspicion of PCa (elevated prostate-specific antigen (PSA), positive Digital Rectal Evaluation, genetic susceptibility) with a standardized PI-RADS V2 score. In the compiled cohort, patients were randomly selected in order to have a large distribution of PI-RADS scores and prostate volumes.

### MRI protocol

MRI exams were performed using a 3 T clinical system (SIGNA™ Architect, GE Healthcare, Chicago, IL and MAGNETOM™ Skyra, Siemens Healthcare, Erlangen, Germany) using a 32-channel phased-array torso coil. Patients were advised to perform bowel preparation before the exam and to empty their bladder; 1 mg glucagon was administered intramuscularly to reduce peristaltic motion. All MRI protocols included 3D T2W images (characteristics of the acquisition are presented in Additional file 1), and for a subgroup of 12 patients, a supplementary axial 2D T2W acquisition.

### Image analysis

#### MRI manual segmentation

**Fig. 1:**
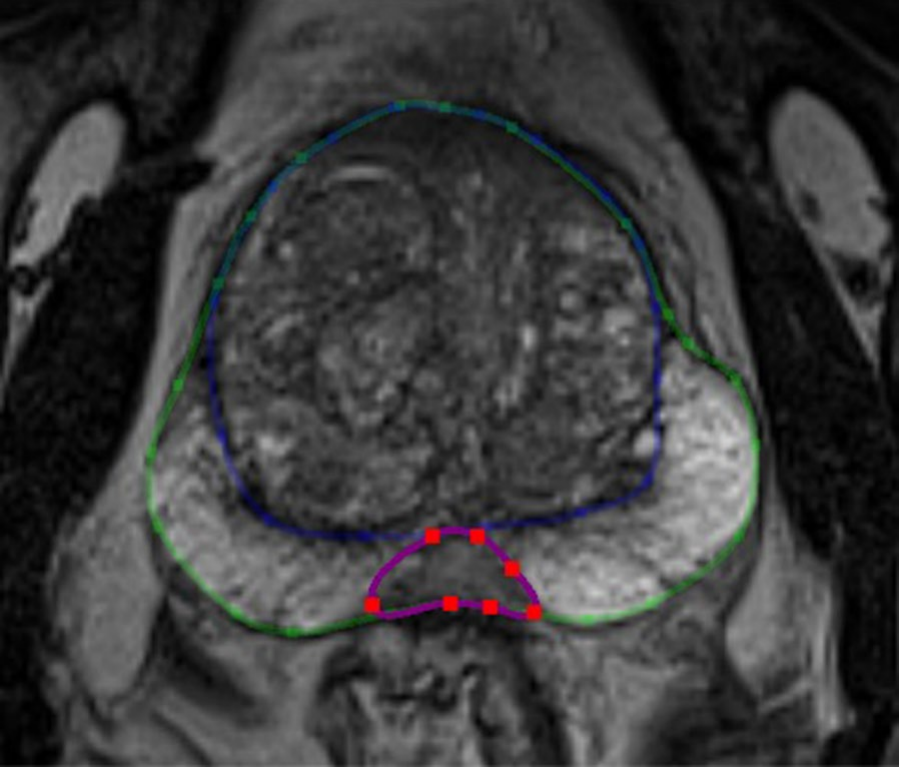
Example of anatomic zonal segmentation. The central zone (purple) is included in PZ (green contour minus blue contour), and not in TZ (blue) on this slice

Seven radiologist readers performed manual segmentation: 3 experts (> 1000 prostate MRI interpreted, G1), 2 seniors (500 prostate MRI, G2) and 2 juniors (< 100 prostate MRI, G3)). A training meeting with the 7 readers was organized before the beginning of the study in order to reach an agreement on segmentation criteria. The basic zonal anatomy of the prostate was reviewed (especially base and apex limits, and the distinction between the TZ and PZ at the base). The readers were instructed to segment the whole gland (WG) and then the transition zone (TZ) first on the axial plane of the 3D T2W sequence (*n* = 40) and then for a sub group of patients on the axial 2D T2W images (*n* = 12). The PZ was obtained by subtracting the WG and the TZ. The CZ and AFMS were not segmented separately for two reasons. The first was that PCa originating in the CZ is uncommon, and because there are no guidelines regarding delineation of the CZ, which is mostly posterior to the TZ, we chose to include it in the PZ. Second, PCa does not originate from the AFMS which is an entirely non glandular zone. Most suspicious lesions in the AFMS arise in the TZ, therefore we considered the AFMS to be part of the TZ. Examples of anatomic zonal segmentation are provided in Fig. 1 and Additional file 2.

Segmentation was performed using MedInria, an open-source software developped by the Inria Research Institute (https://med.inria.fr/). Polygons were delineated on the axial plane of the 3D (*n* = 40) and 2D (*n* = 12) T2W sequences, from the lowest part of the apex to the extreme base: approximately one in every six slices on the 3D T2W images (between 35 and 75 polygons per prostate) and one in every three slices on 2D T2W images. The software performed an interpolation between these polygons to create the whole segmentation. All contours were then carefully checked using MedInria’s capability for visualization in three dimensions (axial, sagittal and coronal) and modified if necessary with a repulsor tool or by directly moving one vertex of the polygon (Additional file 3).

#### Signal intensity and volume measurement

Two readers placed similar sized ROIs in the TZ and in the PZ to evaluate TZ (SI_TZ_) and PZ (SI_PZ_) signal intensity, and then the squared contrast between both was calculated as (SI_TZ_ – SI_PZ_)^2^/(SI_TZ_ + SI_PZ_)^2^.

Each rater provided an estimation of the prostate volume for each MRI, based on the ellipsoid formula: *V* = length * width * height * 0.52 and the mean of these results was used as the prostate volume.

#### Metrics and statistical analysis

We used the open-source software VISCERAL Evaluate Segmentation (Apache License v2) for computation of the metrics used for the comparisons (https://github.com/Visceral-Project/EvaluateSegmentation) and SimpleITK [17, 18]. Two methods were used to evaluate the similarity of the segmentations: pairwise calculation (by comparing each mask one by one, and then considering the mean and the standard deviation of the metrics to compare both readers) and consensus comparison based on STAPLE algorithm [19] (computation of a consensus between the seven raters’ segmentations and calculation of the metrics comparing the masks and the consensus mask generated with SimpleITK [17, 18]).

Because of correlations existing between those metrics, we only performed statistical tests on some of the most commonly used in the literature: The Dice Score (DSC), the Hausdorff Distance (HD), and the Average Hausdorff Distance (AHD) [20]. All metrics are in 3D unless stated otherwise.

To investigate the segmentation variability along the cranio-caudal axis we computed HD and DSC for each third of the prostate: apex, mid-gland and base, taking as limits the upper and lower slices of the masks for the pairwise comparison, and the limits of the consensus mask for the STAPLE comparison. The paired Wilcoxon signed-rank test and Mann–Whitney *U* test were used respectively for related samples and independent samples comparisons. The Spearman correlation ρ was used for the correlation calculations. The *p*-values were corrected with the Holm-Bonferroni method. All statistical tests were two-sided. A *p*-value < 0.05 was considered indicative of a statistically significant difference. We used the Python modules statsmodels (version 0.11.1, www.statsmodels.org) to perform the Holm-Bonferroni correction, and Pingouin (version 0.3.7, pingouin-stats.org) to compute the other statistical tests.

## Results

### Patients

The demographic, biologic and morphologic data for our population are summarized in Table 1. Median age at MRI was 64 years [range 45–76 years], mean PSA level was 8.4 ± 5.6 ng/mL, and median prostate volume was 57.8 cm^3^ [range 15–199]. Among the 40 patients, 17 (42.5%) were classified with a PI-RADS ≥ 3.

**Table 1:** Demographic and clinical characteristics of study participants (*n* = 40)

Variable	Value
Age (years)^a^	64 [45–76]
PSA (ng/mL)^b^	8.4 (± 5.6)
MRI equipment	
3 T SIGNA™ Architect, General Electrics	11 (27%)
3 T MAGNETOM™ Skyra, Siemens Healthcare	29 (73%)
Prostate Volume (cm^3^)^a,c^	57.8 [15–199]
PI-RADS	
PI-RADS 1–2	23 (57.5%)
PI-RADS 3	4 (10%)
PI-RADS 4	6 (15%)
PI-RADS 5	7 (17.5%)
Tumor location evaluated on MRI	
Peripheral zone (PZ)	10 (25%)
Transitional zone (TZ)	7 (17%)

^a^Median [range]

^b^Mean (± STD)

^c^Median volume was estimated by the median of all the volumes the readers estimated from the MRIs, using an ellipsoid formula

### Inter-reader variability of prostate segmentation: WG verssu TZ

#### Pairwise comparison

When evaluating the WG, we obtained a mean DSC of 0.92 (± SD = 0.02), a mean HD of 9.8 (± 3.8) voxels, and a mean AHD of 0.17 (± 0.08) voxels.

**Fig. 2:**
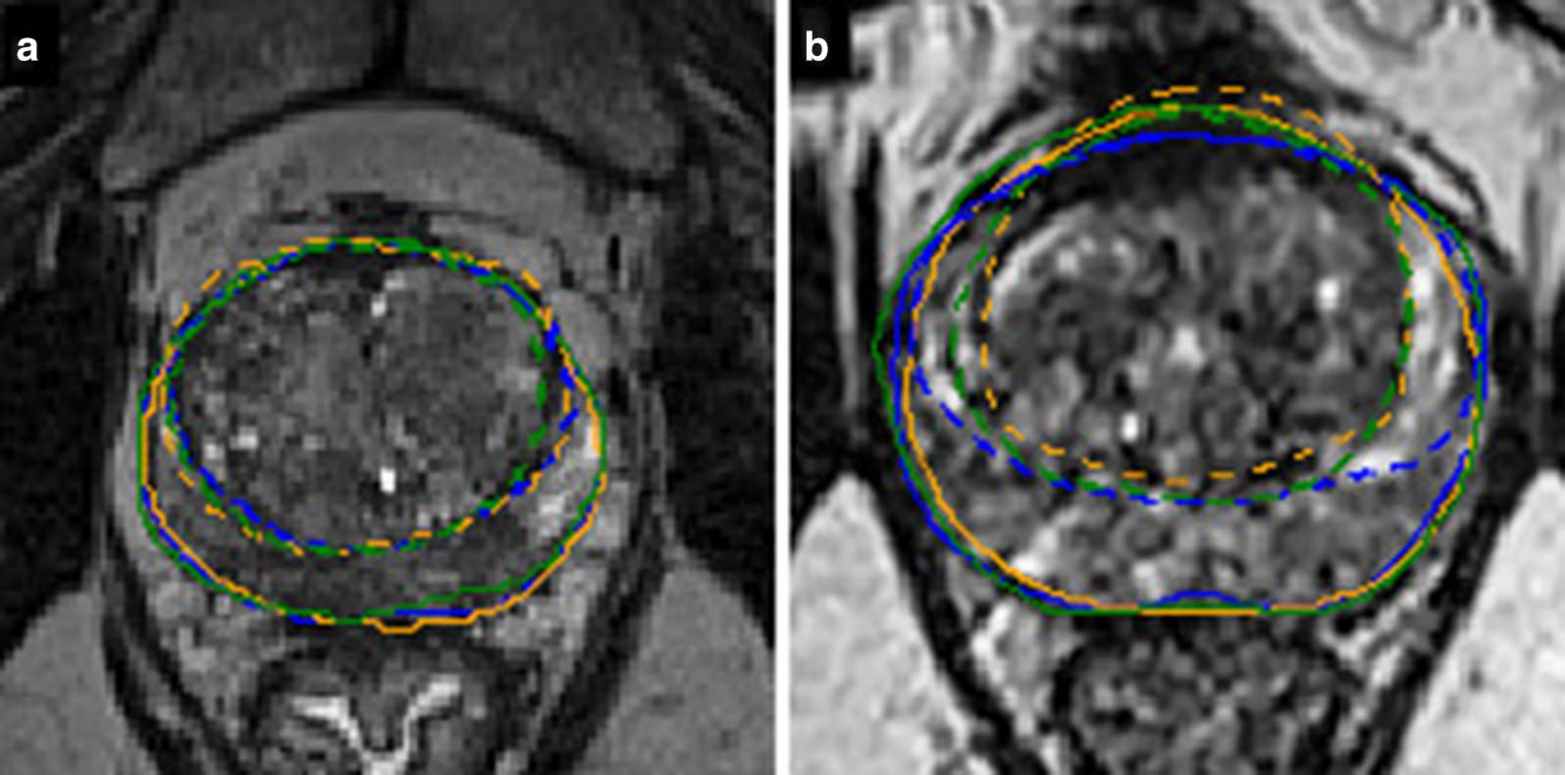
Examples of low (**a**) and high (**b**) segmentation variabilities for WG (full line) and TZ (dashed line) on a transverse slice for one rater of each group of experience (blue for expert, orange for senior, green for resident)

**Fig. 3:**
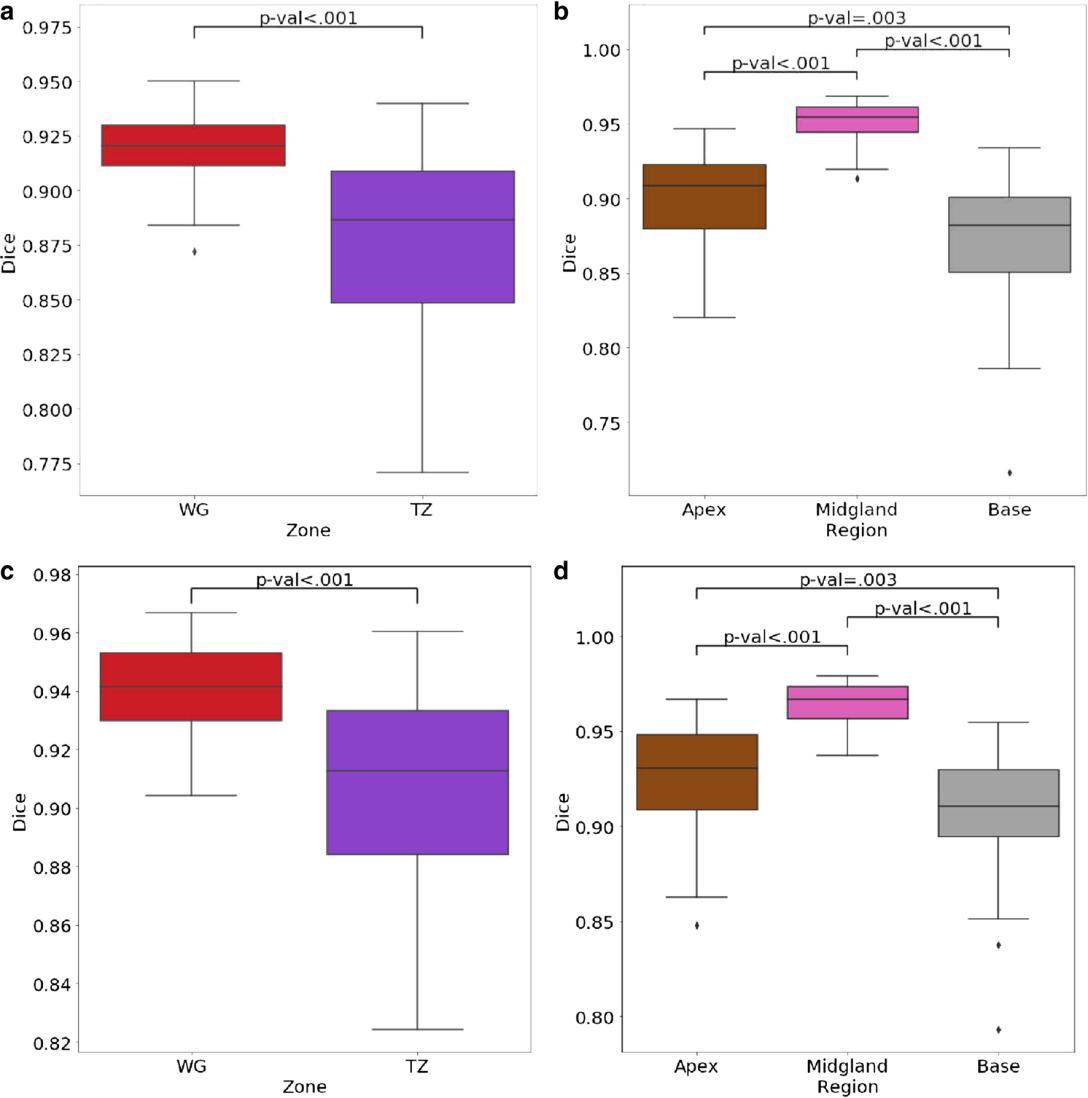
Comparison of DSC for segmentations of WG and TZ (**a**, **c**), and for WG segmentation when the prostate is divided along the cranio-caudal axis in the base/mid-gland/apex (**b**, **d**), using a pairwise comparison (**a**, **b**) and a consensus comparison (**c**, **d**)

Concerning the TZ we found a higher variability with a mean DSC of 0.88 (± SD = 0.05), an increase of the mean HD to 12.0 (± 4.9) voxels, and an increase of the mean AHD to 0.31 (± 0.19) voxels. An example of segmentation variability between the different readers groups is shown in Fig. 2, and the global results are illustrated in Fig. 3.

#### Consensus comparison (STAPLE method)

Results (summarized in Table 2) were similar for the WG with a mean DSC of 0.94 (± SD = 0.03), a mean HD of 8.15 (± 3.33) voxels and a mean AHD of 0.11 (± 0.07) voxels, and a higher variability for TZ with a mean DSC of 0.91 (± SD = 0.05), a mean HD of 10.0 (± 4.2) voxels, and a mean AHD of 0.21 (± 0.16) voxels.

**Table 2:** Summarized similarity metrics for all radiologists and all structures (WG vs. TZ, and WG divided along cranio-caudal axis in base, mid-gland and apex), with 2 methods (pair-wise comparison and consensus comparison (STAPLE reference))

Structure	Method	DSC	HD (voxels)	AHD (voxels)
WG	Pairwise	0.92 ± 0.02	9.77 ± 3.78	0.17 ± 0.08
TZ		0.88 ± 0.05	11.98 ± 4.92	0.31 ± 0.19
WG	STAPLE	0.94 ± 0.03	8.15 ± 3.33	0.11 ± 0.07
TZ		0.91 ± 0.05	10.03 ± 4.25	0.21 ± 0.16
Base	Pairwise	0.87 ± 0.06	9.66 ± 4.61	
Mid-gland		0.95 ± 0.02	7.51 ± 3.63	
Apex		0.90 ± 0.06	7.12 ± 3.73	
Base	STAPLE	0.91 ± 0.06	7.87 ± 3.69	
Mid-gland		0.96 ± 0.02	6.10 ± 3.05	
Apex		0.93 ± 0.05	5.88 ± 3.05	

### Inter-reader variability of prostate segmentation: regions/cranio-caudal axis

With the pairwise method the lowest similarity was found at the base with a mean DSC and HD respectively of 0.87 (± SD = 0.06) and 9.66 (± 4.61) voxels, compared to the apex (mean DSC and HD respectively of 0.90 (± 0.06), and 7.12 (± 3.72) voxels), and to the mid-gland (mean DSC and HD respectively of 0.95 (± 0.02) and 7.51 (± 3.63) voxels). All comparisons between the base and other regions were found to be significant for both metrics. Similar results were obtained with the STAPLE method. These results are summarized in Table 2 and illustrated in Fig. 3.

### Inter-reader variability of prostate segmentation: impact of prostate morphological differences

We found that the smaller the prostate was, the higher the variability was (using DSC for both methods), *ρ* > 0.8 (*p*-value < 0.001).

A low squared TZ to PZ contrast was significantly associated with a higher segmentation variability (*ρ* = 0.5 (CI 95% = [0.23; 0.7], *p*-value = 0.01 and 0.45 (CI 95% = [0.17; 0.67], *p*-value = 0.03) for the pairwise method and the consensus comparison (STAPLE method).

**Fig. 4:**
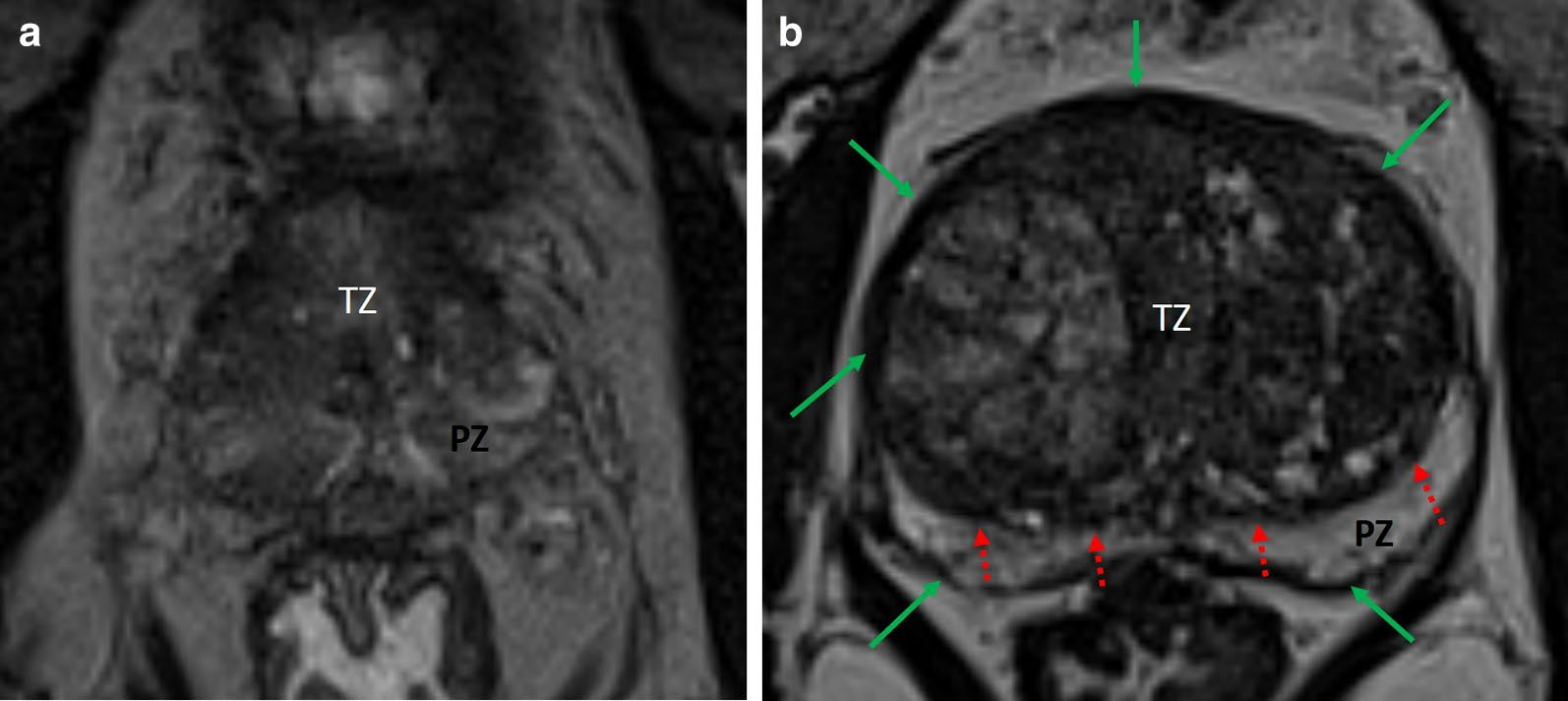
Influence of variation in prostate volume on zonal differentiation. **a** Poor zonal differentiation in a small prostate volume (20 cm^3^); **b** Clear zonal anatomy differentiation in a larger prostate volume (120 cm^3^): pseudo-capsule (green arrows) and TZ delimitation (red dotted arrows) are clearly individualizable

**Fig. 5:**
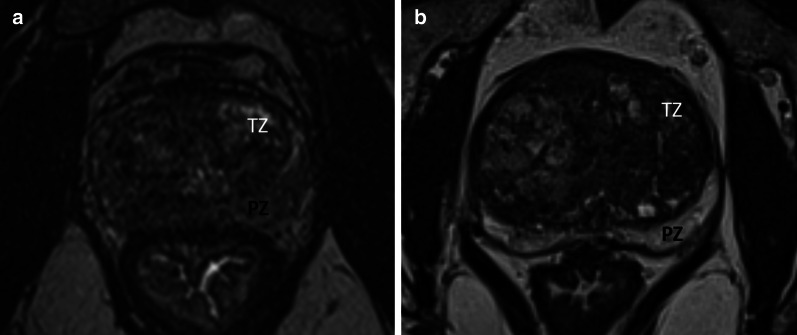
Influence of intensity signal ratio between the TZ and the PZ on zonal differentiation, **a** Moderate signal difference between zones (signal ratio = 0.98); **b** Marked difference in signal intensity, facilitating zonal differenciation (signal ratio = 0.37)

No significant difference was found when considering the impact of the presence of tumor (*p*-value = 0.53 for the mean DSC on the WG). Finally, a retro-urethral lobe protruding into the bladder showed no significant influence on segmentation variability (*p*-value = 0.08 for the mean DSC on the WG). These results are detailed in Table 3 and illustrated in Figs. 4, 5 and Additional file 4 and Additional file 5.

**Table 3:** Impact of various factors on segmentation variability with 2 methods (pair-wise comparison and consensus comparison (STAPLE reference))

		Pair-wise comparison			STAPLE reference		
Factor	Metric	*ρ*^a^	CI 95%	*p*-value	*ρ*^a^	CI 95%	*p*-value
Volume^a^	DSC	0.84	[0.72; 0.91]	< 0.001	0.86	[0.76; 0.93]	< 0.001
	HD (voxels)	0.28	[− 0.04; 0.54]	0.5	0.20	[− 0.12; 0.48]	1.0
	AHD (voxels)	− 0.42	[− 0.65; − 0.13]	0.06	− 0.63	[− 0.78; − 0.39]	< 0.001
Squared TZ to PZ contrast^a^	DSC	0.50	[0.23; 0.71]	0.01	0.45	|0.17; 0.67]	0.03
	HD (voxels)	− 0.03	[− 0.34; 0.29]	1.0	− 0.04	[− 0.34; 0.28]	1.0
	AHD (voxels)	− 0.49	[− 0.7; − 0.22]	0.01	− 0.45	[− 0.67; − 0.17]	0.03
Presence of a PI-RADS ≥ 3 lesion^b^	DSC			0.53			0.71
	HD (voxels)			0.3			0.47
	AHD (voxels)			1.0			1.0
Large median lobe^b^	DSC			0.08			0.11
	HD (voxels)			0.61			1.0
	AHD (voxels)			1.0			1.0

^a^Test: spearman correlation

^b^Test: Mann-u-Whitney

### Inter-reader variability of prostate segmentation: impact of reader expertise

Masks from the 3 different groups of radiologists (expert, senior, and junior) were compared to the consensus (STAPLE reference).

**Fig. 6:**
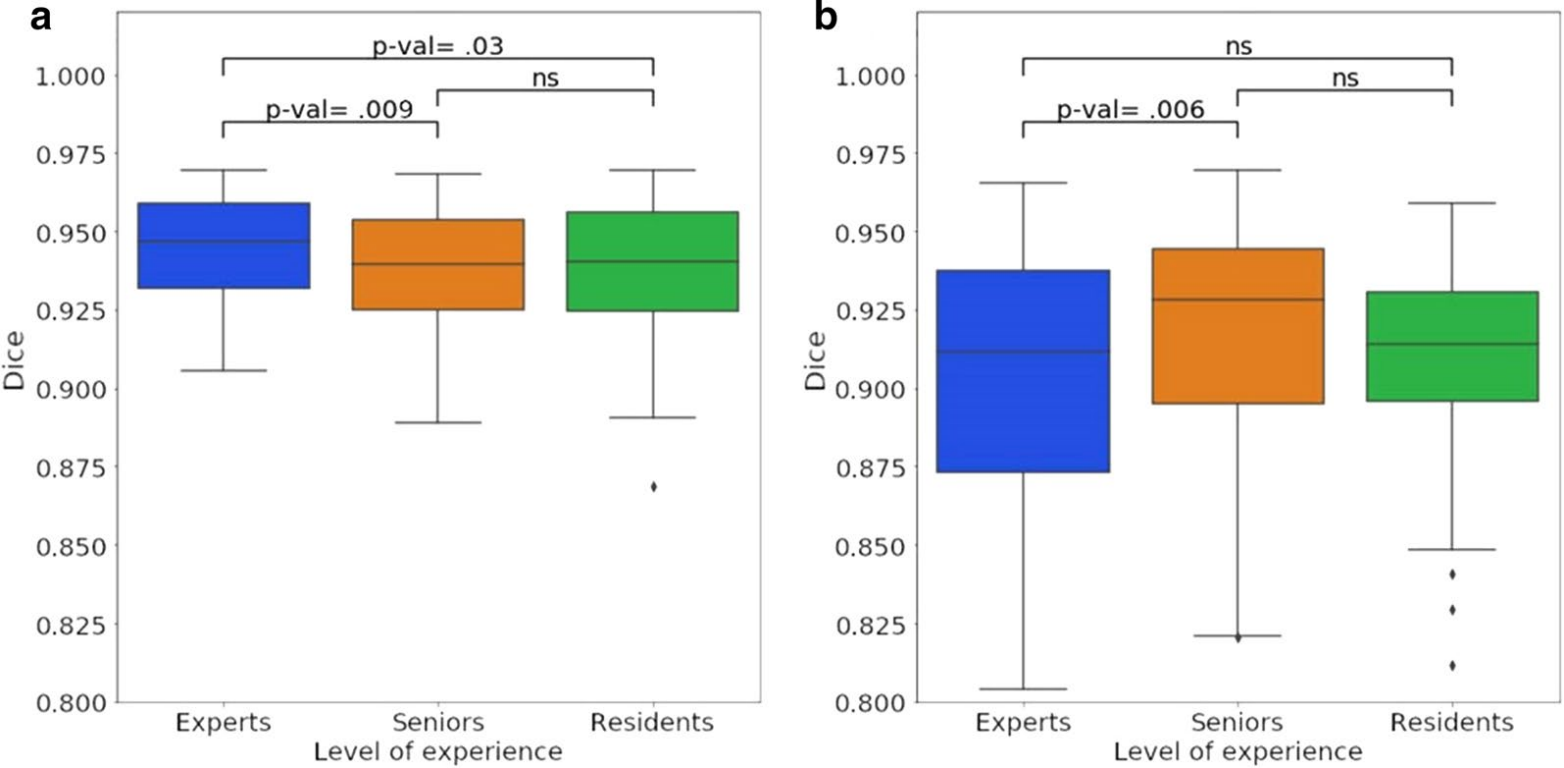
Impact of the readers’ level of expertise (expert/senior/resident) on segmentation variability evaluated by DSC, for WG (**a**) and TZ (**b**) segmentation (ns = not significant)

For WG, G1, G2, and G3 had respectively a mean DSC of 0.944 (± 0.023), 0.936 (± 0.031), and 0.938 (± 0.025). G1 was the closest to the consensus (*p*-value = 0.009 and 0.03 for G1/G2 and G1/G3 comparison) (Fig. 6). Similar results were obtained using HD and AHD.

On TZ, G1, G2, and G3 had respectively a mean DSC of 0.903 (± 0.061), 0.916 (± 0.055), and 0.907 (± 0.04). G2 was the closest to the consensus but was not significantly closer than G3 (*p*-value = 0.27). The results are summarized in Table 4.

**Table 4:** Segmentation variability according to the reader’s level of expertise (3 experts/2 seniors/2 residents), with their comparison’ associated *p*-values

Structure	Group	DSC	*p*-value versus seniors	*p*-value versus residents	HD (voxels)	*p*-value versus seniors	*p*-value versus residents	AHD (voxels)	*p*-value versus seniors	*p*-value versus residents
WG	Experts	0.944 ± 0.023	0.09	0.03	7.74 ± 3.15	1.15	0.14	0.10 ± 0.05	0.03	0.009
	Seniors	0.936 ± 0.031	–	0.91	8.49 ± 3.47	–	1.0	0.12 ± 0.08	–	1.0
	Residents	0.938 ± 0.025	0.91	–	8.42 ± 3.41	1.0	–	0.13 ± 0.08	1.0	–
TZ	Experts	0.903 ± 0.061	0.01	1.0	10.50 ± 4.60	0.007	1.0	0.22 ± 0.18	0.002	1.0
	Seniors	0.916 ± 0.055	–	0.27	8.93 ± 3.66	–	0.01	0.17 ± 0.16	–	0.09
	Residents	0.907 ± 0.040	0.27	–	10.42 ± 4.12	0.01	–	0.22 ± 0.13	0.09	–

### Inter-reader variability: 2D versus 3D segmentation

Results are summarized in Table 5.

**Table 5:** 2D versus 3D T2W MRI segmentation variability (*n* = 12)

Structure	DSC			HD (mm)		
	2D	3D	*p*-value	2D	3D	*p*-value
**Zone**^a^						
WG	0.90 ± 0.03	0.91 ± 0.03	0.006	6.97 ± 2.54	6.68 ± 2.06	0.24
TZ	0.86 ± 0.06	0.86 ± 0.06	0.8	7.92 ± 3.07	7.53 ± 2.23	0.44
**Region**^b^						
Base	0.70 ± 0.16	0.71 ± 0.15	0.32	6.89 ± 2.79	6.31 ± 2.2	0.03
Mid-gland	0.94 ± 0.02	0.95 ± 0.02	0.01	4.14 ± 1.11	4.31 ± 1.59	0.33
Apex	0.76 ± 0.16	0.79 ± 0.12	0.11	4.54 ± 1.69	4.23 ± 1.56	0.05*

^a^Computations with 3D metrics

^b^Computations with slicewise metrics

^*^Not significant

#### WG versus TZ

No significant difference was shown when comparing segmentation on 3D T2W versus segmentation on 2D T2W, neither with DSC for the TZ with 0.860 versus 0.861 (*p*-value = 0.8), nor with HD on WG and TZ (*p*-value = 0.24 and 0.44 respectively). The only exception was the mean DSC for WG with 0.91 vs 0.90 (*p*-value = 0.006).

#### Cranio-caudal axis

We found higher mean slicewise DSC and HD for 3D versus 2D MRI segmentations, but these differences were statistically significant only for mid-gland DSC and base HD (*p*-value = 0.01 and 0.03).

## Discussion

Manual delineation of the internal structure of the prostate performed by human experts is the main approach for generating the ground truth in order to develop automated PCa diagnosis algorithms. Very few studies have investigated the variability of the manual zonal prostate delineation, and for automated segmentation tools under development, a quality and well-described ground truth is rarely available.

To identify sources of variability that may influence the quality of the ground truth for the development of automatic zonal segmentation of the prostate gland, we evaluated in this study the influence of reader expertise, variation of prostate morphology, and in a subgroup of patients variability due to images acquisition differences.

We found a low variability when evaluating the WG (DSC of 0.92 and 0.94 with pairwise and STAPLE method respectively) and slightly higher variability for the TZ segmentation (DSC of 0.88 and 0.91). In the cranio-caudal axis we found a lower similarity at the base (DSC 0.87) and the apex (DSC 0.90) of the prostate.

To our knowledge, two studies have evaluated the inter-reader variability of the zonal anatomy [16, 21]; Becker et al. [16] found in a multi-reader study (2 experts radiologists, 2 residents, and 2 computer vision scientists), in a cohort of 80 patients using a 3.0 T MRI and endo-rectal coil, a DSC of 0.733 for the WG and a higher variability for the TZ (DSC 0.738), in the apex (2D DSC 0.85) and basal part of the gland (2D DSC 0.87). Padgett et al. [22] in a multi-reader study (*n* = 2) of zonal segmentation on 2D T2W images obtained on 3 T of 30 consecutive patients found for the WG a DSC of 0.88 ± 0.04 and 0.81 ± 0.1 for the TZ.

Our results are partly in line with these previously published studies and highlight the difficulty of zonal segmentation especially at the ends of the gland: (a) the apex that has an intensity profile similar to surrounding structures, fuzzy borders, and poor image contrast at the boundary, and (b) at the base with the tricky challenge of partial volume effect between the PZ and the TZ.

Unlike those two previous studies we have chosen to include the CZ in the PZ segmentation.

There are no current guidelines in particular regarding whether the CZ should be delineated separately or included in the PZ or in the TZ, and figures provided in the different studies do not clearly indicate this specific point. The CZ, which appears as a symmetric band of tissue between the peripheral and the transition zones at the base of the prostate, extending from below the seminal vesicles to the verumontanum, is extremely difficult to delineate, because it is usually compressed and displaced. Very few cancers arise from this area, (around 7%) [23], and even in the PI-RADS score [9], there is no guidance on how to derive the PI-RADS assessment category for such lesions involving the CZ. It is suggested that CZ lesions should receive PI-RADS score as if they were located in the zone from which they are most likely to be coming from (the PZ or TZ) [9, 24]. This highlights the need to work on guidelines for prostate delineation for the development of automatic tools.

We evaluated the influence of expertise with 7 readers of varying experiences divided into 3 groups. We did not find any substantial difference on TZ and WG segmentation. Results were statistically significant but numerical DSC values were very close (0.94 for the 3 groups) and showed no substantial difference. Our overall segmentation variability scored higher than those previously published whatever the region analyzed and the level of expertise. However, all readers in our study were radiologists and have benefited from a training meeting before the start of the study, in order to precisely define the segmentation criteria. This is concordant with the results of Becker et al. [16], who only found significant differences between non-radiologists and radiologists and concluded that inter-reader baseline of non-radiologists may not be sufficient for meaningful comparison to new segmentation algorithms.

Previous studies emphasize the challenge of automated segmentation because of variation in prostate size and shape but there is not description of such variability in the databases, and no evaluation of the influence of anatomical variations such as prostate volume, intensity contrast ratio between TZ and PZ, or the presence of visible lesions.

The prostate gland is a complex organ with varied size, shape and appearance. Morphological differences may contribute to segmentation variability. We found that the smaller the prostate, the higher the segmentation variability was (*p* < 0.001). Hyperplasia of the TZ leading to prostate hypertrophy is the most common change attributed to aging [25]. We hypothesized that the increase in size of TZ was associated with sharper contours (surgical capsule) which are then easier to draw, whereas in small volume prostate without prostatic hyperplasia, the glandular tissues of the transition and peripheral zone are histologically identical [25] and therefore more difficult to differentiate. In our cohort, 42.5% of MRIs had a PI-RADS score > 2 with lesions in both the PZ and the TZ that may alter the appearance of anatomical structure under segmentation. The presence or absence of a PI-RADS score > 2 lesion did not translate into an increase in segmentation variability (*p* = 0.53). However, the variability increased with a lower PZ to TZ contrast ratio (*p*-value = 0.01) which can be explain by poor contrast at boundary between zones.

Variation in image acquisition such as 3D versus 2D T2W images could translate into variability of segmentation. Unlike 2D T2W, the 3D T2W sequences are acquired with sub-millimeter resolution, to allow the acquisition of a volume that can be reconstructed into any plane with an improvement of anatomic delineation. Although we are aware of the limited number of patients, we didn’t find any substantial differences in the subgroup (*n* = 12) who benefited from both types of acquisition.

Zonal prostate segmentation is a fundamental step in the development of automated PCa diagnosis algorithms. In the PROMISE12 challenge [4] reference segmentations of the WG were provided in each center by an experienced reader, and were checked by a second expert (with more than 1000 prostate MRIs analyses) who was asked to correct the potential WG segmentation inconsistencies. The resulting segmentation was used as the reference standard and served as a training set for the development for multiple AI algorithms. However, the PROMISE12 database does not provide any zonal information of the prostate besides the WG and furthermore relies only on a single reference standard. Yet, the estimation of inter-observer variability is very important to assess the practical performance of an algorithm with respect to human experts. Indeed, this variability reflects the intrinsic ambiguity of the segmentation task, and an algorithm performance can be properly assessed by testing whether its output falls within the range of inter-observer variability. Knowledge of the factors influencing the quality of prostate zonal segmentation may also contribute to producing high-quality labeled training data essential for PCa detection and PI-RADS score application. Well-defined guidelines to ensure consistency and accuracy of manual delineation of the prostate are currently not available and should be developed and followed to generate ground truth segmentations. To account for the anatomical and disease-related variability among different patients, as well as the variability in image acquisition, image databases should include representative clinical samples with anatomical variation and patients with different tumors according to their localization.

Some limitations can be found in our study. First, the number of cases processed was limited (*n* = 40). However, this is because manual segmentation is an extremely time consuming process, and we partly compensated for this with a relatively high number of raters, who segmented 52 MRIs each (40 3D and 12 2D T2W MRI), and 2 statistical methods with similar results. We found only one study [16] with more cases segmented (80 vs. 40) but fewer readers (6 vs. our 7). Second, technical differences between data sets such as the use of 3D T2W and a pelvic coil instead of an endo-rectal coil also represent a difficulty for comparison to previous studies. However, most prostate MRIs are now realized with a surface coil. Finally, we should also point out the lack of non-radiologist readers, which would have been interesting as it was discussed by Becker et al. [16] to evaluate the impact of expertise.

## Conclusion

Identifying sources of variability of prostate zonal segmentation that may influence the quality of the ground truth is a prerequisite for the development of automated PCa detection algorithms. In this study we found that segmentation variability was higher in the extreme parts of the gland, influenced by change in prostate morphology such volume and intensity ratio between zones and was not substantially influenced by radiologist’s expertise. This highlights the need to include representative clinical samples with morphological variation in image databases.

## Supplementary Information

**Additional file 1. Table 1**: MRI acquisition specificities (2 MRI equipments were used for the cohort) (.doc)

**Additional file 2. Figure 1**: Example of superimposed segmentation masks on the corresponding T2W: PZ (+CZ) is blue, TZ (+AFMS) is yellow. (.eps)

**Additional file 3. Figure 2**: Example of a segmentation with manually drawn polygons (thick lines visible on TZ) and result of the interpolation between them (thin lines), reformat in a coronal plan. TZ is drawn in blue, WG is drawn in green.

**Additional file 4: Figure 3**. Relationship between variability (evaluated by DSC) and prostate volume for WG segmentation, using pairwise comparison (.eps)

**Additional file 5: Figure 4**. Example of prostate tumor modifying zones contours. a: PIRADS 5 tumor in the PZ (blue arrow); b: PIRADS 5 tumor in the TZ, with contour deformation (red arrows) (.jpg)

## Data Availability

Data were extracted from the Clinical Data Warehouse of the Greater Paris University Hospitals (Assistance Publique – Hôpitaux de Paris).

## Abbreviations

AFMS: Anterior fibromuscular stroma
AHD: Average Hausdorff distance
CZ: Central zone
DSC: Dice score
G1: Group1
G2: Group2
G3: Group3
HD: Hausdorff distance
PCa: Prostate cancer
PSA: Prostate-specific antigen
PZ: Peripheral zone
SI_PZ_: Mean signal intensity for PZ
SI_TZ_: Mean signal intensity for TZ
T2W: T2-weighted
TZ: Transition zone
WG: Whole gland

## Declarations

### Guarantor

The scientific guarantor of this publication is Pr Raphaële Renard Penna.

### Statistics and biometry

One of the authors has significant statistical expertise: Dimitri Hamzaoui.
