# Supplementary material for: Challenge of prostate MRI segmentation on T2-weighted images: inter-observer variability and impact of prostate morphology
Source: Insights Imaging. 2021 Jun 5;12:71. doi: 10.1186/s13244-021-01010-9 (PMC8179870; doi:10.1186/s13244-021-01010-9)
Supplement: Supplementary file 1 — Additional file 1. Table 1: MRI acquisition specificities (2 MRI equipments were used for the cohort) (.doc) [file 13244_2021_1010_MOESM1_ESM.doc]

**ELECTRONIC SUPPLEMENTARY MATERIAL**

**Supplementary Material 1 : MRI acquisition specificities**

|  | 3T SIGNA^TM^ Architect, GE Healthcare, Chicago, IL* | | 3T MAGNETOM^TM^ Skyra, Siemens Healthcare, Erlangen, Germany** | |
| --- | --- | --- | --- | --- |
| Parameter | Axial T2WI | 3D T2WI | Axial T2WI | 3D T2WI |
| Sequence type | FSE | Echo de spin Cube | TSE | SPACE |
| Field of view (mm) | 200 | 280 | 250 | 230 |
| Acquisition matrix | 512x512 | 512x512 | 296x334 | 230x320 |
| Repetition time (ms) | 9861 | 1602 | 3050 | 1550 |
| Echo time (ms) | 153.14 | 102.87 | 84 | 173 |
| Flip angle (degrees) | 170 | - | 133 | 115 |
| Slice thickness (mm) | 2.5 | 1 | 2.5 | 0.85 |
| Image reconstruction matrix (pixels) | 0.7x0.9x2.5 | 0.8x0.8x1 | 0.7x0.7x2.5 | 0.4x0.4x0.85 |
| Time for acquisition (min:s) | 3min34 | 5min11 | 3min14 | 5min35 |

*Receiver frequency coils: 16-channel phased array body small coil and 32-channel spine coil.

^**^Receiver frequency coils: 18-channel phased array body coil and 32-channel spine coil.

T2WI = T2-weighted imaging, FSE = Fast Spin Echo, TSE = turbo spin-echo, SPACE = Sampling Perfection with Application optimized Contrasts using different flip angle Evolution
