# Supplementary figures and images for: Challenge of prostate MRI segmentation on T2-weighted images: inter-observer variability and impact of prostate morphology
Source: Insights Imaging. 2021 Jun 5;12:71. doi: 10.1186/s13244-021-01010-9 (PMC8179870; doi:10.1186/s13244-021-01010-9)

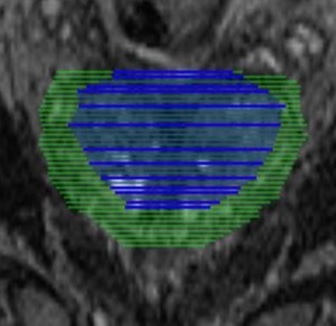

Supplement: Supplementary file 3 — Additional file 3. Figure 2: Example of a segmentation with manually drawn polygons (thick lines visible on TZ) and result of the interpolation between them (thin lines), reformat in a coronal plan. TZ is drawn in blue, WG is drawn in green. [file 13244_2021_1010_MOESM3_ESM.jpg]

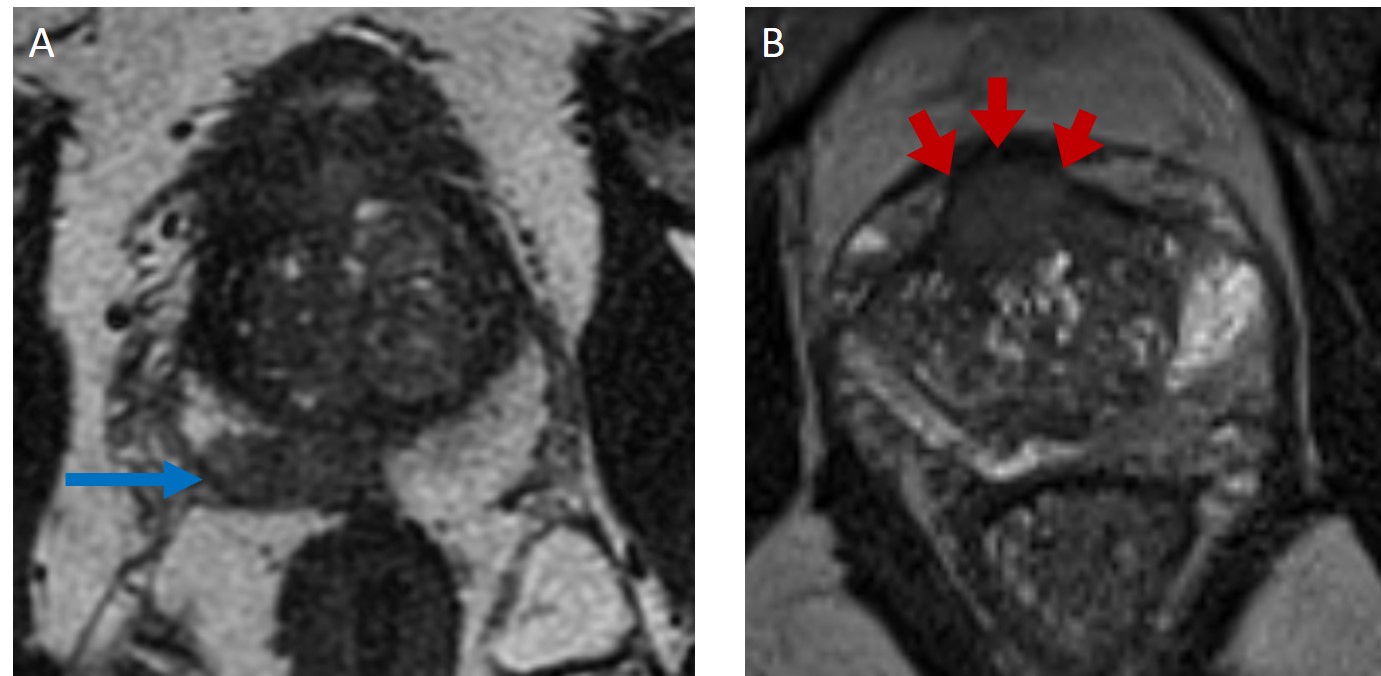

Supplement: Supplementary file 5 — Additional file 5: Figure 4. Example of prostate tumor modifying zones contours. a: PIRADS 5 tumor in the PZ (blue arrow); b: PIRADS 5 tumor in the TZ, with contour deformation (red arrows) (.jpg) [file 13244_2021_1010_MOESM5_ESM.jpg]
